# Supplementary material for: Identifying a Vaginal Microbiome-Derived Selective Antibiotic Metabolite via Microbiome Pharmacology Analysis
Source: bioRxiv. 2025 Aug 28:2025.08.28.672927. Preprint. [Version 1] doi: 10.1101/2025.08.28.672927 (PMC12407940; doi:10.1101/2025.08.28.672927)
Supplement: Supplementary file 1 [file NIHPP2025.08.28.672927v1-supplement-1.pdf]

bioRxiv preprint doi: https://doi.org/10.1101/2025.08.28.672927; this version posted August 28, 2025. The copyright holder for this preprint (which was not certified by peer review) is the author/funder, who has granted bioRxiv a license to display the preprint in perpetuity. It is made available under aCC-BY 4.0 International license.

Figure S1.

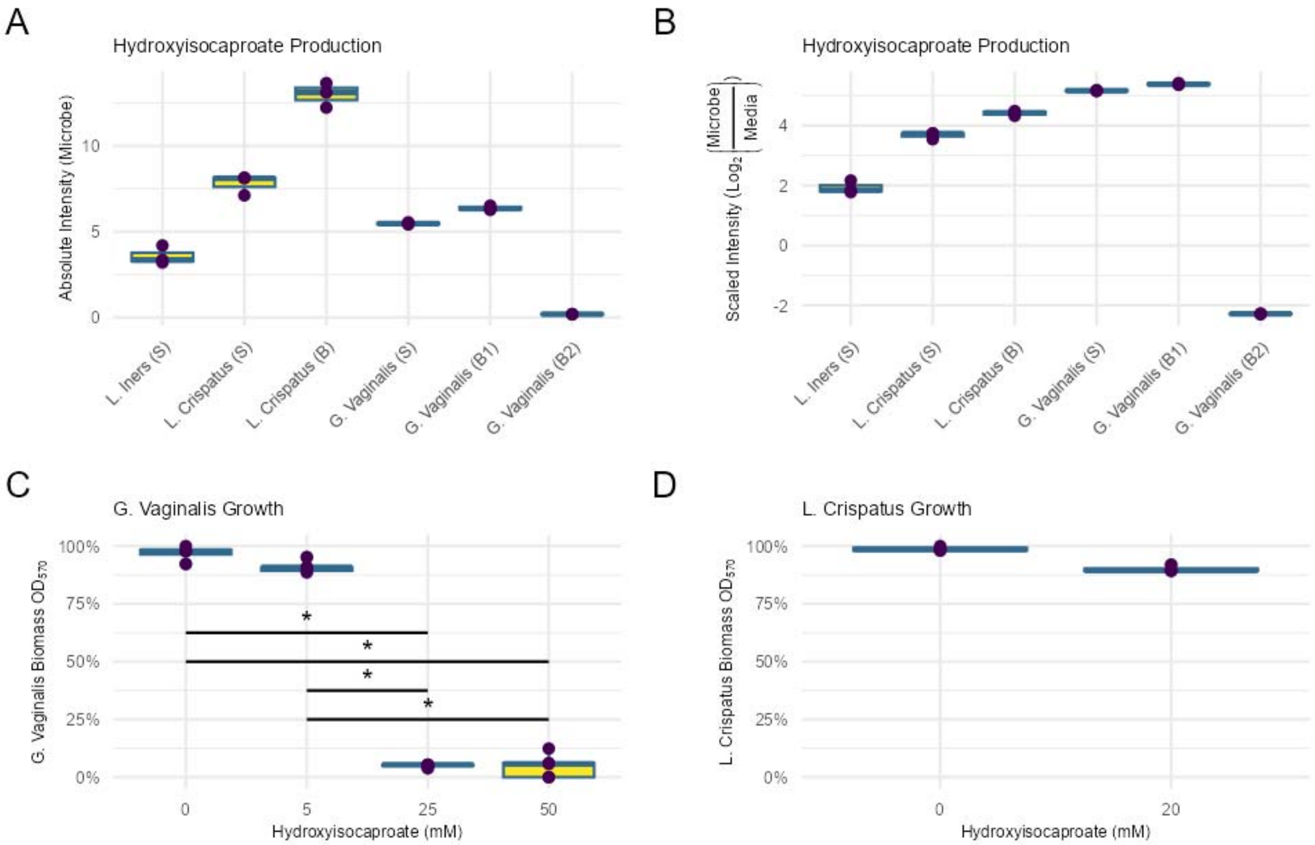

Figure S1. Functional characterization of the antibiotic properties of Hydroxyisocaproate (HICA) (A) HICA production by isolated, cultured type strains of vaginal bacteria in suspension (S) and biofilm (B) conditions. *G. vaginalis* biofilms were grown in NYCIII (B1) or supplemented BHI (B2) media. **(B)** HICA production scaled to media **(C)** *G. vaginalis* growth inhibition by HICA treatment. **(D)** *L. crispatus* growth inhibition by HICA treatment.
